# What do we really know about brucellosis diagnosis in livestock worldwide? A systematic review

**DOI:** 10.1371/journal.pntd.0013185

**Published:** 2025-06-17

**Authors:** Sonia Vection, Christopher G. Laine, Angela M. Arenas-Gamboa

**Affiliations:** Department of Veterinary Pathobiology, College of Veterinary Medicine and Biomedical Sciences, Texas A&M University, College Station, Texas, United States of America; Institute of Continuing Medical Education of Ioannina, GREECE

## Abstract

Diagnosis of brucellosis is not a straightforward task, with over 40 different tests available. Accurate diagnosis requires a series of diagnostic testing with proper interpretation of results. The World Organization for Animal Health (WOAH) provides guidelines describing the different assays including their recommended use, protocols, and interpretation. PubMed, Embase and Web of Science databases were searched without restrictions and original work describing cross-sectional studies focusing on livestock species (cattle, buffaloes, sheep, goats and swine) were included while reviews, case reports, and case-control studies were excluded. In this study, we systematically reviewed the literature and critically assessed the findings from 349 research studies to provide an overview of the different diagnostic methods used worldwide in livestock, and compared the tools and strategies used against the WOAH recommendations. A total of 232 studies (66.5%) focused on cattle followed by goats (34.1%), sheep (31.5%), buffaloes (14.6%), and swine (5.2%). Of these studies, 171 were from Africa (48.9%), 132 from Asia (37.8%), 36 from the Americas (10.3%), and 10 from Europe (2.8%). The most utilized immunological assayswere Rose Bengal test and indirect ELISA (63.9 and 36.7%, respectively). Interestingly, 73 studies (20.9%) used a single immunological assay to report on the status of animals. Direct methods such as culture and PCR were performed in 100 studies (28.7%) with culture being the most utilized (19.8%). Strikingly, we found that only 16% of included studies followed WOAH recommendations in terms of sample chosen, diagnostic assay utilized, protocol employed and results interpretation. In countries that reported the presence of *B. abortus*, *B. melitensis*, and *B. suis* to the WOAH, only 4 of 28, 2 of 19, and 1 of 6 countries (reporting these strains, respectively), contained studies that followed guidelines and confirmed the presence of the pathogen. This highlights, not only significant gaps in currently available literature leading to an inaccurate picture of brucellosis in livestock, but most importantly raises significant issues regarding the accuracy of data reported by countries. These findings are concerning due to the significant consequences of not adhering to these guidelines including inaccurate diagnosis, delayed disease control, and increased zoonotic risk for exposed individuals.

## Author summary

Brucellosis is a zoonotic bacterial disease that can affect domestic and wild animals worldwide. Diagnosis of the disease is not straightforward and usually requires several tests to be conducted for an accurate conclusion. Guidelines for recommended use of assays are provided by the World Organization for Animal Health (WOAH) which describe the experimental protocols and interpretation of the results for each test. However, the vast number of available assays and the numerous ways they can be combined make it challenging to gain a clear understanding of brucellosis epidemiology worldwide. This systematic review provides an overview of the different diagnostic tests and strategies used worldwide for the diagnosis of brucellosis in livestock. Experimental methods and interpretations were analyzed and compared with WOAH recommendations to assess compliance with their guidelines in an effort to provide a better understanding of the epidemiology of the disease worldwide.

## Introduction

Brucellosis is a zoonotic disease caused by bacteria of the *Brucella* genus. Among the 12 recognized species, there are 3 most virulent to livestock and animals, and are known to be endemic worldwide with the exemption of Canada, Japan, Australia, New Zealand, and several countries in Europe [1–4]. These species are *Brucella abortus* (which primarily infects cattle), *Brucella melitensis* (most commonly found in sheep and goats), and *Brucella suis* (mainly infecting swine). Animals typically acquire the bacteria through an oropharyngeal route of transmission after contact with infected materials such as placental tissues, aborted fetuses, and milk, which are known to contain high numbers of the organism [5]. Signs of the disease in animals are mostly reproductive with abortions and stillborn offspring [6]. However, these signs are not pathognomonic to brucellosis as many other pathogens can also induce abortions [7]. The disease is not limited to animals as the bacterium can be transmitted to humans, mainly through the consumption of unpasteurized dairy products and contact with infected reproductive tissues, causing a newly estimated 1.6 to 2.1 million human cases per year [8].

Despite recent attempts to determine the number of new brucellosis cases per year both in animals and humans, the actual number remains unknown. To a certain extent, this can be attributed to the fact that the diagnosis of brucellosis is not a straightforward task, requiring a series of diagnostic testing coupled with a proper understanding and interpretation of test results. To help circumvent this problem, the World Organization for Animal Health (WOAH) (formerly OIE) has, over the years, provided publicly available guidelines in their “Manual of Diagnostic Tests and Vaccines for Terrestrial Animals” [9]. This document outlines the different diagnostic assays suitable for animals infected with *B. abortus*, *B. melitensis,* or *B. suis*, and provides information on the assay’s purpose and recommended usage. It also describes the correct methodologies for sample collection, choice of diagnostic test, reagents standardization, and experimental protocols to ensure proper diagnosis. These guidelines serve as recommendations, and their success relies on how researchers, veterinarians, or public health professionals implement them based on the disease knowledge, the individual country needs, and the local disease status. In this study we aim to 1) provide an overview of the different diagnostic methods currently being utilized in the diagnosis of brucellosis in livestock worldwide, 2) compare the diagnostic methods utilized by researchers worldwide against WOAH guidelines, and 3) correlate the findings of research studies that followed WOAH recommendations with the self-reported country level data posted on the World Animal Health Information System ( WAHIS). It is our expectation that these findings will provide insight on the state of the existing knowledge concerning the epidemiology of brucellosis in livestock and highlight the strengths and opportunities in disease diagnosis with the intention of providing evidence-based information that will guide future research and policies within this area.

## Results

### Search results

**Fig 1:**
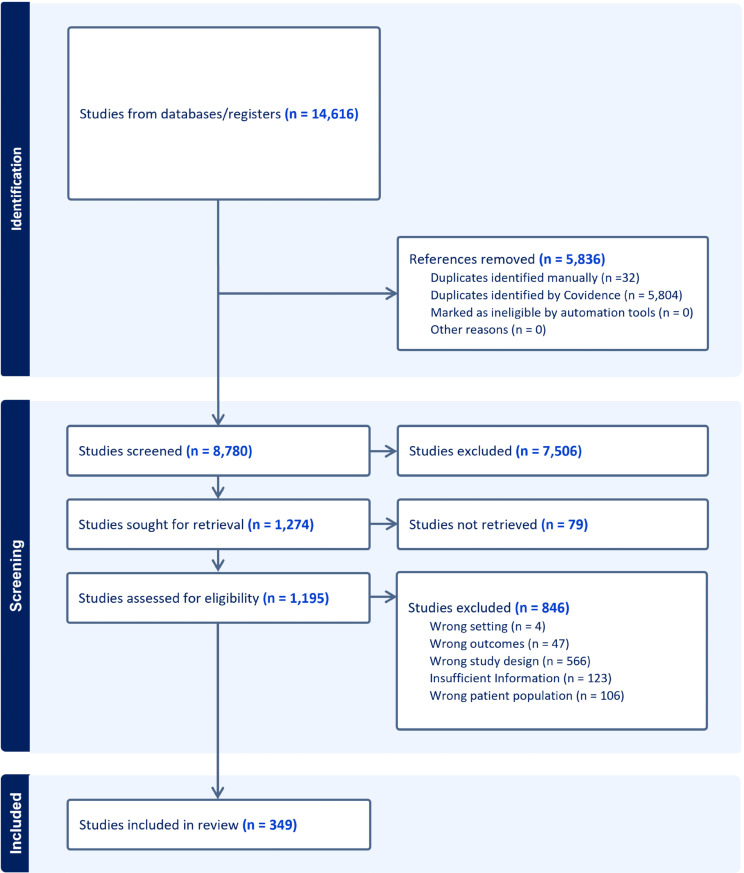
PRISMA flow diagram for the selection of the studies analyzed.

The employed search strategy retrieved 14,616 research studies, among which 5,836 were duplicates and subsequently removed either by the Covidence software or manually. The remaining studies were screened for title and abstract, resulting in the exclusion of 7,506 additional studies based on their study type, target population, and/or overall objective. From the remaining 1,274 studies, 79 were unavailable, leaving 1,195 studies to be assessed for eligibility. After full text screening, 846 studies were excluded for the following reasons: inappropriate study design (566), insufficient information (123), incorrect subject population (106), different outcomes (study focusing on *Brucella* species other than *B. melitensis*, *abortus*, or *suis*) (47), or wrong setting (4). Consequently, 349 articles were included in this study for data extraction (Fig 1).

**Fig 2:**
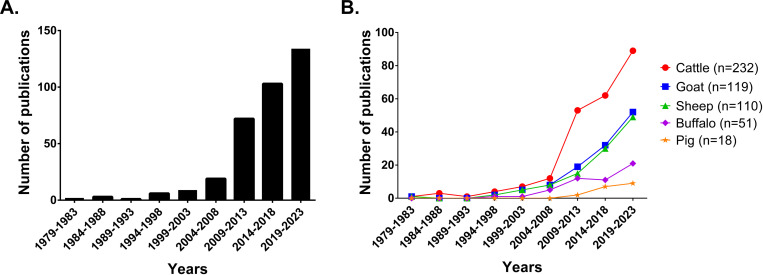
Temporal distribution and frequency of included studies by 5 years intervals from 1979 to 2023. (A) Overall temporal distribution. (B) Temporal distribution by livestock species studied.

A total of 349 studies, published between 1979 and 2023, were within the scope of this study. Interestingly, the number of publications drastically increased in the last 14 years, between 2009 and 2023, with 309 studies published during this time frame (Fig 2). Globally, most of the studies sampled cattle (232/349, 66.5%) (Fig 2), followed by goats (119/349, 34.1%), sheep (110/349, 31.5%), buffaloes (51/349, 14.6%) and swine (18/349, 5.2%) indicating that cattle were the most studied species with almost twice the number of studies when compared to small ruminants. Early research focused mainly on cattle, while most studies focusing on small ruminants and buffaloes started after 1994 with all swine investigations being published after 2009.

**Fig 3:**
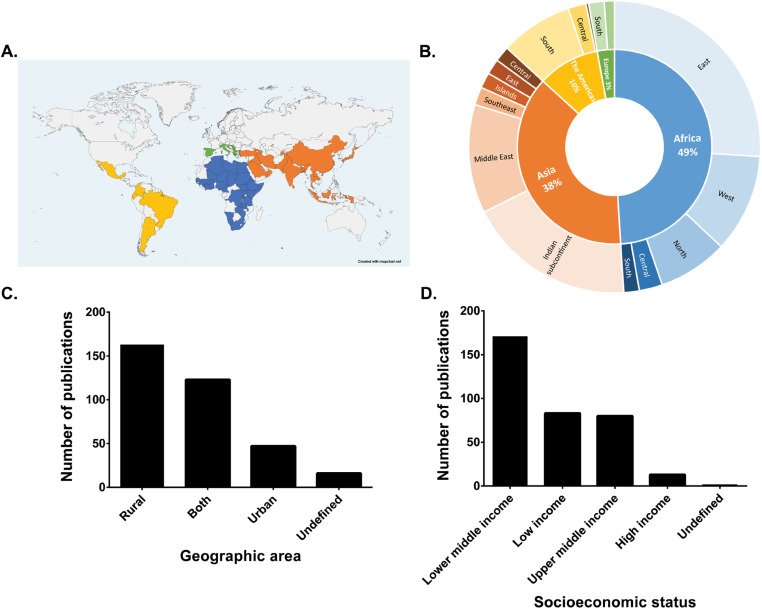
Geographical distribution, area characteristics (urban or rural) and socioeconomic status. (A) Geographical distribution of the selected studies focusing on the diagnosis of brucellosis in livestock. Colored countries were included in the study. The map was created using mapchart.net and the basemap is available at: https://www.mapchart.net/world.html. (B) Repartition of the selected studies by continent and subregion. (C) Country classification based on the area characteristic of the sampled region. (D) Distribution of countries by socioeconomic status.

Studies were globally distributed and originated in Africa, The Americas, Asia, and Europe, with Oceania and Antarctica lacking representation (Fig 3). Almost half of the studies (171/349, 48.9%) originated in Africa, primarily from the eastern and western regions. Additionally, 37.8% (132/349) were conducted in Asia, with the majority (65/349, 49.2%) taking place in the Indian subcontinent. Research performed in the Americas (36/349, 10.3%) was focused within South and Central America while no study was recovered from North America. Interestingly, only 2.8% (10/349) of the studies were conducted in Europe, with emphasis on the southern region.

The socioeconomic status of each country was subsequently evaluated, and countries were placed into designated groups based on the GNI per capita of each country utilizing the publicly available information from the World Bank [10,11]. Countries were categorized as low-income, lower middle-income, upper middle-income, and high-income economies (S3 Fig). Interestingly, most of the studies (254/349, 72.8%,) were conducted in low and lower-middle income countries. Specifically, of the 349 studies, 171 (171/349, 49%) originated in lower middle countries, 83 (83/349, 23.8%) in low-income countries, 80 (80/349, 22.9%) in upper middle countries, and only 13 (13/349, 3.7%) in high income countries (Fig 3C). Two (2/349, 0.6%) of the studies were focused on a regional basis rather than on a particular country, and, therefore, the economic status was not determined and subsequently categorized as undefined.

Once the geographical location and socioeconomic status were assessed, the precise location of each study was extracted to determine the geographic environment of the sampled area (rural, urban, both, or undefined). This parameter is especially important as brucellosis is more prevalent in rural areas where most of the livestock is located. As expected, the majority of studies were conducted in rural settings (163/349, 46.7%) while 47 (47/349, 13.5%) were conducted in urban areas. Sixteen studies (16/349, 4.6%) did not provide information of the precise location of the sampled region and were classified as undefined (Fig 3D). The remainder of the studies (123/349, 35.2%) included a combination of both rural and urban areas.

Most of the studies sampling cattle were located in Africa (131/232, 56.5%), followed by Asia (82/232, 35.3%), the Americas (15/232, 6.5%), and Europe (4/232, 1.7%) (S1 Table). Studies describing *Brucella* infection in buffaloes were most common in Asia (40/51, 78.4%), followed by the Americas (8/51, 15.7%), Africa (2/51, 3.9%), and Europe (1/51, 2%). For small ruminants, the number of studies in Africa and Asia were very similar, with 52 (52/110, 47.3%) studies including sheep as the subject population in Africa and 50 (50/110, 45.5%) in Asia. Furthermore, 59 (59/119, 49.6%) and 46 (46/119, 38.7%) studies investigated goats in Africa and Asia, respectively.

### Diagnostic test usage and interpretation

Our next objective was to provide an overview of the different assays used for the diagnosis of brucellosis in livestock globally. Immunological testing (indirect method) was clearly the preferred approach used in 310 (310/349, 88.8%) of the studies. Overall, the most used immunological assay was RBT (223/349, 63.9%) followed by iELISA (128/349, 36.7%), and CFT (70/349, 20.1%). Within these studies, the majority (249/349, 71.3%) relied exclusively on serology for the diagnosis and did not include additional direct diagnostic methods such as culture or PCR. Only half of the studies (176/349, 50.4%) utilized at least two immunological tests to determine the disease status. The most used combination was RBT and CFT (39/349, 11.2%) which was mostly utilized in Africa followed by RBT and iELISA (36/349, 10.3%) which was most common in Asia, while RBT and cELISA (29/349, 16.5%) were mostly used in Africa. Overall, we identified 38 different combinations of immunological tests. In addition, 73 studies (73/349, 20.9%) utilized a single immunological assay to report on the infection status of animals. When using a single test, iELISA (30/73, 41%) was preferred, especially in Asia (Table 1).

**Table 1:** Ten most used testing methodologies for the diagnosis of brucellosis in livestock by continental region or worldwide.

	Number of publications				
Testing Methodology	Worldwide	Africa	Asia	Europe	The Americas
RBT and CFT	39	33	3	1	2
RBT and iELISA	36	13	20	1	2
iELISA	30	8	17	1	4
RBT and cELISA	29	24	4	0	1
RBT	22	15	4	0	3
PCR and Culture	15	3	9	0	3
cELISA	11	8	0	0	3
RBT and SAT	12	7	5	0	0
PCR	7	0	4	1	2
RBT, iELISA, PCR, and Culture	6	1	5	0	0
Culture	5	0	5	0	0

RBT: Rose Bengal test, CFT: Complement Fixation Test, iELISA: indirect enzyme-linked immunosorbent assay, cELISA: competitive enzyme-linked immunosorbent assay, SAT: Serum Agglutination Test, PCR: Polymerase Chain Reaction.

Only 100 (28.6%) of the studies utilized direct methods to diagnose brucellosis. Direct methods include bacteriological culture and direct detection of the organism’s genetic material via PCR. These approaches were used alone (36/100, 36%) or in combination with immunological testing (64/100, 64%). Isolation of the pathogen by culture was only performed in 68 (68/349, 19.5%) studies using different tissue samples including lymph nodes, fetal lung and abomasal contents, placenta, liver, spleen, hygroma fluid and reproductive organs (47/68, 69.1%), milk (21/68, 30.9%), vaginal swabs (17/68, 25%), and blood (9/68, 13.2%). Identification of *Brucella* species was also done by PCR (55/349, 16%) and biotyping (56/349, 16%), followed by qPCR (26/349, 7.4%). Other approaches included MLVA (6/349, 1.7%), sequencing (5/349, 1.4%), and mass spectrometry (5/349, 1.4%). *Brucella abortus* was the most commonly identified species, detected in 59 studies (59/349, 16.9%), mostly isolated in Asia (31/59, 52.5%) followed by Africa (21/59, 35.6%), the Americas (6/59, 10.2%), and Europe (1/59, 1.7%). *B. melitensis* was identified in 46 studies (46/349, 13.2%) with the majority in Asia (30/46, 65.2%) followed by Africa (13/46, 28.3%), Europe (2/46,4.3%), and the Americas (1/46, 2.2%). *B. suis* was only detected in 9 studies (2.6%), mainly in Asia and Europe (3/9, 33.3%) followed by Africa (2/9, 2.2%) and the Americas (1/9, 11.1%). Surprisingly, a total of 100 different test combinations including direct and indirect methods were used in the analyzed studies, clearly demonstrating a significant lack of consensus on the diagnostic approach for brucellosis detection in livestock.

### Comparison of methods with WOAH guidelines

The next objective was to better understand the methodology utilized within the different studies to provide insight on the use and interpretation of diagnostic testing. For this, the diagnostic assays and the diagnostic approach in each study (which included reagents, protocols and samples) were evaluated and compared with WOAH recommendations.

Concerning immunological assays, the RBT was mostly performed as recommended (163/223, 73.1%) with only 12 studies (12/223, 5.4%) not following recommendations (Table 2). The remainder of the studies (48/223, 21.4%) did not provide sufficient information to determine if the protocol utilized followed recommendations. The majority of studies that included the use of indirect ELISA were not in accordance with guidelines (114/128, 89.1%). The most common differences with guidelines during assay performance were the absence of validation of the assay using local samples from target populations (114/128, 89.1%), the utilization of kits that were not designed for the specific samples being tested (3/128, 2.3%), and the use of kits not standardized according to requirements (1/128, 0.8%). Only one study (1/128, 0.8%) reported validating the assay using local controls samples calibrated against WOAH standards. The remainder of the studies did not include sufficient details to allow for a conclusion (53/128, 41.4%). CFT was used in 70 studies but only 84.3% (59/70) of them provided enough information to conclude that the protocol followed was in accordance with WOAH guidelines. The rest of the studies did not provide details of the methods used which made conclusions impossible (10/70, 14.3%). Strikingly, another test worth mentioning is the modified mercaptoethanol (2-ME) tube agglutination assay which is based on the addition of beta-mercaptoethanol in the reaction to breakdown IgM pentamers and reduce false positive results. However, this test is only recommended by WOAH for the diagnosis of *Brucella canis* in dogs and not for use in livestock. This assay was inadequately used as a “confirmatory” test in 13 (13/349, 3.7%) studies. A similar issue was observed with the Rivanol test. Similarly to the 2-ME test, the addition of a rivanol solution selectively precipitates IgMs in the serum. It was used in 3 (3/349, 0.9%) studies as a “confirmatory” assay although this test is not recommended in the guidelines. Therefore, results from studies using both of these tests should be deemed inconclusive or unreliable.

**Table 2:** Classification of protocols utilized in research studies with respect to agreement with recommendations made by the WOAH.

Test performed	Performed according to WOAH recommendations	Not performed according to WOAH recommendations	Insufficient information	Total number of publications
RBT	163	12	48	223
iELISA	1	114	13	128
CFT	59	1	10	70
Culture	51	8	9	68
Tissues	36	2	9	47
Milk	15	6	0	21
Vaginal swabs	14	3	0	17
Blood	8	1	0	9
cELISA	1	52	7	60
PCR	44	10	2	55
Tissues	8	1	1	10
Isolates	33	4	0	37
Blood	0	2	0	2
Serum	0	3	0	3
Milk	3	0	0	3
Paraffin-embedded tissues	0	0	1	1
Biotyping	51	2	4	57
SAT	17	28	4	49
qPCR	8	18	0	26
Tissues	1	1	0	2
Isolates	5	2	0	7
Blood	0	6	0	6
Vaginal swab	0	1	0	1
Milk	3	2	0	5
Cream and yogurt	1	0	0	1
Serum	0	9	0	9
MRT	12	0	4	16
Plate agglutination test	2	1	0	3
2-ME	0	7	6	13
mRBT	8	1	0	9
MLVA	5	1	0	6
LFA	0	2	3	5
Sequencing	2	1	2	5
MS	0	4	1	5
FPA	2	0	1	3
Rivanol	0	3	0	3
BPAT	5	0	4	9
Double gel immunodiffusion	1	0	0	1
Dot Blot Assay	0	1	0	1

Samples used were subjected to PCR, qPCR and bacteriological culture. RBT: Rose Bengal test, iELISA: indirect enzyme-linked immunosorbent assay, CFT: Complement Fixation Test, cELISA: competitive enzyme-linked immunosorbent assay, PCR: Polymerase Chain Reaction, SAT: Serum Agglutination Test, qPCR: quantitative PCR, MRT: Milk Ring Test, 2-ME: 2-Mercaptoethanol, mRBT: modified Rose Bengal Test, MLVA: Multiple Locus Variable-Number Tandem Repeat Analysis, LFA: Lateral Flow Assay, MS: Mass Spectrometry, FPA: Fluorescence Polarization Assay, BPAT: Buffered Plate Agglutination Test.

It is well established that bacteriological culture is the gold standard for the definitive diagnosis of brucellosis as it allows for the isolation of the pathogen. However, it was only used in 68 studies (68/349, 19.5%) and only performed as recommended in 75% (51/69) of cases. The main issues were the improper selection of growth media (2/6, 33.3%) and sample preparation protocol (4/6, 66.7%). Another method that can be used for the detection of *Brucella* in tissue samples is PCR. This test was conducted in a total of 55 studies (55/349, 15.8%) among which 19 (19/55, 34.5%) used PCR on different types of samples such as tissues, blood, serum, and milk. Overall, this test was performed as recommended in 58% (11/19) of the studies focusing on tissue samples. The main pitfalls included the use of serum (4/6, 66.7%) and blood (2/6, 33.3%) as targeted samples and the improper selection of primers (2/6, 33.3%). Two studies (2/19, 10.5%) did not include details of the procedure utilized. Finally, real-time PCR was done in 26 (26/349, 7.4%) studies among which 19 (19/26, 73.1%) focused on samples such as tissues, blood, serum, vaginal swabs, milk, and yogurt. Overall, this assay was not conducted in accordance with available guidelines in 16 out of 19 studies (16/19, 84.2%). The main pitfalls included the use of serum (8/16, 50%) and blood (6/16, 37.5%) as samples, as well as the inadequate choice of primers (2/16, 12.5%).

Following bacterial isolation, confirmation of the genus *Brucella* was conducted in 63 (63/349, 18.1%) studies and was primarily achieved using biotyping which relies on lysis by phages and/or the oxidative metabolic profiles of *Brucella* species. This method was performed in 56 studies (56/349, 16%) with most of the studies (50/56, 89.3%) following recommendations. Two studies (2/56, 3.57%) used protocols different from the recommendations. The remainder of the studies (6/56, 10.7%) did not provide sufficient information to allow for a conclusion. Another method used for identification of *Brucella* was MVLA. As *Brucella* genomes are highly similar, tandem repeat analysis targets repeating regions that were shown to differ between species [12]. This technique goes beyond species identification and can be used to understand the phylogenetic relationships between samples, offering deeper insights in epidemiology. This test was used in 6 (6/349, 1.7%) studies and was performed according to guidelines in 5 (5/7, 83.3%) of them. One study did not follow recommendations, using an improper number of targets (1/6, 16.7%). *Brucella* identification was also done using PCR based techniques in 37 studies (37/349, 10.6%). Two of the most commonly described conventional multiplex PCR assays are the *Brucella* AMOS PCR and the Bruce Ladder [9]. Both allow for the identification of *B. abortus* (wild type and vaccine strains S19 and RB51), *B. melitensis*, *B. suis*, and *B. ovis* while the Bruce Ladder also includes *B. canis*, *B. neotomae*, *B. ceti*, *B. pinnipedialis* and the vaccine strain *B. melitensis* Rev 1 [13–15]. The AMOS PCR was used in 16 studies (16/55, 29.1%) (S2 Table). Among these, 14 (14/16, 87.5%) studies employed this approach on isolates, and all were performed according to WOAH recommendations (14/14, 100%). Two studies were classified as not in accordance with WOAH guidelines as one (1/19, 5.3%) used serum samples and the other (1/19, 5.3%) used tissue samples. The Bruce Ladder was used in 7 (7/55, 12.7%) reports that targeted isolates (S2 Table). None of the studies attempted to use tissue samples. The protocol was performed according to guidelines in all studies (7/7, 100%). Finally, 3 reports (3/55, 5.5%) utilized both AMOS PCR and Bruce Ladder on isolates according to WOAH recommendations (3/3, 100%). Taken together, these results suggest that a significant portion of assays performed to diagnose brucellosis in livestock or identify *Brucella* after isolation were not carried out according to the recommended standards. Interestingly, the data suggests that WOAH guidelines, even though publicly available, might either not be known, understood, or applied especially for molecular approaches, and that efforts in education should aid in enhancing the overall quality of research.

Following the analysis of experimental protocols utilized for each assay, we subsequently focused our efforts on test result interpretation in each study and the type of conclusions that were drawn from such interpretations. Culture is the gold standard for the diagnosis of brucellosis as it provides a definite diagnosis of the disease, and the interpretation of results is straight forward. However, this is not the case for immunological assays, mainly due to the known limitations of such assays (see discussion section below). Therefore, the use of multiple immunological tests might be warranted based on the objective of testing and the interpretation of such assays be done in relation to each other rather than independently. To do so, diagnostic strategies such as “series testing” or “parallel testing” can be utilized but results should be interpreted carefully especially in countries where vaccination is performed due to the inability to differentiate vaccinated animals from infected ones using immunological tests. Our analysis revealed that independent interpretation of the results of immunological assays was performed in almost half of the studies (145/349, 41.5%) with the majority in Africa (71/145, 49%) followed by Asia (59/145, 40.7%), the Americas (12/145, 8.3%), and Europe (3/145, 2.1%). Series testing was performed in 151 studies (151/349, 43.3%) mainly in Africa (88/151, 58.3%) followed by Asia (42/151, 27.8%), the Americas (15/151, 9.9%), and Europe (6/151, 4%). Finally, only 14 studies (14/349, 4%) conducted parallel testing in Africa and Asia (6/14, 42.9%) equally, followed by the Americas (2/14, 14.3%). None of the studies focusing on Europe utilized this strategy. These results highlight deficiencies in the interpretation of results, potentially leading to inexact data and increased confusion on the epidemiological status of a given region or country when performing a poorly defined protocol other than recommended by the WOAH.

**Fig 4:**
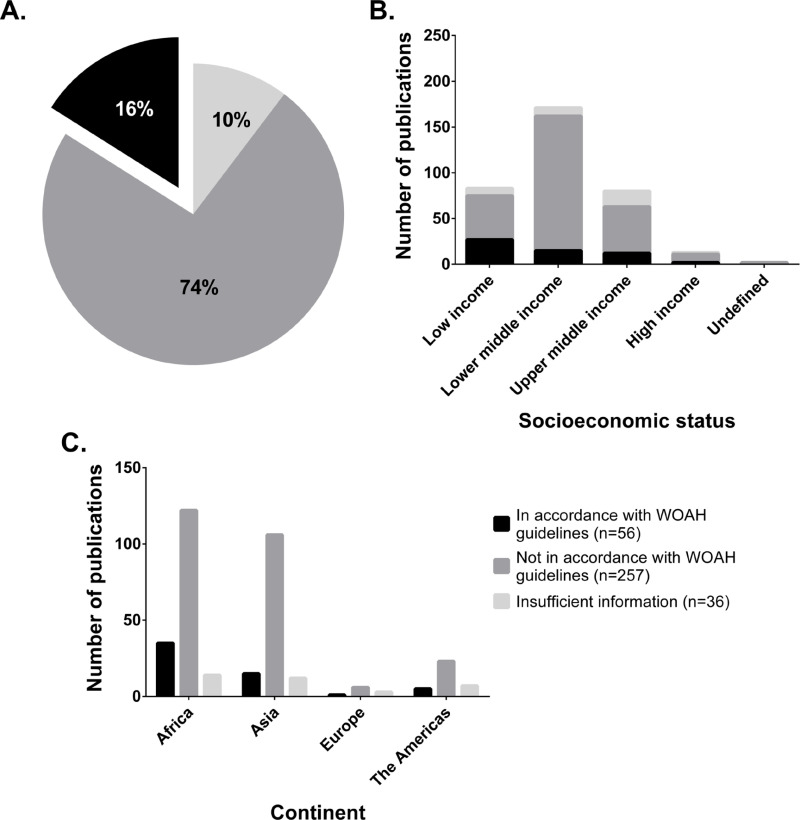
Adherence of studies to WOAH guidelines by socioeconomic status and continent. Sample type, choice of diagnostics tests, applied protocols, and interpretation of results were determined and subsequently classified into 3 groups: 1) Insufficient information (critical information missing that did not allow for a conclusion), 2) Not in accordance (one or several of the mentioned parameters were not in accordance with WOAH recommendations), or 3) In accordance (all of the mentioned parameters were in accordance with WOAH recommendations). (A) Overall classification of studies based on accordance to guidelines. (B) Classification of studies based on accordance to guidelines and socioeconomic status. (C) Classification of studies based on accordance to guidelines and geographical region.

After analysis of the individual parameters of each study (sample type, choice of diagnostic tests, experimental protocols, and results interpretation), studies were classified depending on their overall agreement with WOAH guidelines. This allows for the analysis of studies as a whole and leads to the identification of high confidence studies reporting accurate data. Studies in which all the above-mentioned parameters agreed with standards were classified as in accordance. Strikingly, only 16% (56/349) of the studies were conducted based on available recommendations (Fig 4). The majority of these studies were performed in Africa (35/56, 62.4%) followed by Asia (15/56, 26.8%), the Americas (5/56, 8.9%), and Europe (1/56, 1.8%). Studies in which at least one of the above-mentioned parameters did not agree with recommendations were classified as not in accordance. This was the case for 257 studies (257/349, 73.6%) focusing on Africa and Asia (122/257, 47.5% and 106/257, 41.2%, respectively), followed by the Americas (23/257, 8.9%) and Europe (6/257, 2.3%). The remaining studies (36/349, 10.3%) omitted critical information in the experimental methods that preclude their categorization. These studies mainly focused on Africa (14/36, 38.9%), followed by Asia (12/36, 33.3%), the Americas (7/36, 19.4%) and Europe (3/36, 8.3%). These results highlight a significant gap in the application of publicly available guidelines to accurately diagnose brucellosis in livestock.

To further assess if the lack of adherence was based on the socioeconomic status of a specific region, we subsequently compared each study to the socioeconomic status of the sampled country (Fig 4). In low-income countries, 27 studies (27/83, 32.5%) were in accordance with recommendations while 48 (48/83, 57.8%) were not. The remainder of the studies (8/83, 9.6%) lacked key information in the diagnostic methods utilized and were classified as “insufficient information”. In lower-middle income countries, 15 studies (15/171, 8.8%) were in accordance while 147 (147/171, 86%) were not. The rest of the studies (9/171, 5.3%) were classified as “insufficient information”. In upper-middle income countries, only 12 studies (12/80, 15%) followed guidelines while 51 (51/80, 63.8%) did not. Remaining studies (17/80, 21.3%) did not include sufficient details and were classified as “insufficient information”. Finally, 2 (2/13, 15.4%) of the studies focusing on high income countries followed guidelines whereas 9 (9/13, 69.2%) differed from them. The rest of the studies (2/13, 15.4%) were classified as “insufficient information”. This demonstrates that the lack of adherence to current recommendations is not correlated with the socioeconomic status of a specific region.

### Correlation between officially reported disease status to WOAH vs. research study findings

Brucellosis in livestock is a notifiable disease, and countries are encouraged to report their status regarding the 3 major *Brucella* species in livestock to the WOAH annually. These reports consist of the self-evaluation of the presence or absence of *B. melitensis*, *B. abortus*, and *B. suis* in livestock populations and can be found in the WAHIS. However, no details on diagnostic tests utilized or experimental protocols explaining how these conclusions were drawn are required. Therefore, the quality of these reports cannot be assessed. As an alternative approach, we compared the findings from research studies performed fully according to guidelines to the officially reported country-level data to assess if research findings reflect the data reported by the countries. To do so, we compared reports from the WAHIS database to findings of research studies conducted during the most recent 5-year time frame available (between 2014 and 2018) as reporting significantly decreased during the COVID-19 pandemic, resulting in incomplete datasets in subsequent years. Based on their reports, individual countries were classified as 1) “endemic” if they reported the presence of a given *Brucella* species at least 3 of the 5 years, 2) “disease free” if they actively reported being free from a given species during 3 of the 5 years, and 3) “insufficient information” if they did not share any information during the chosen time period (S3 Table). Between 2014 and 2018, we retrieved 103 studies from 37 different countries in which key parameters analyzed in the previous section were all in accordance with available recommendations.

**Fig 5:**
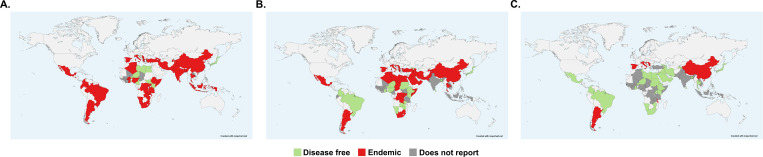
Geographical representation of the presence or absence of the major zoonotic species of *Brucella* as reported to the WOAH. (A) Presence or absence of *B. abortus*, (B) *B. melitensis*, and (C) *B. suis*. Countries considered disease free are labeled in green, while those that are considered endemic are labelled in red. Countries that do not report any information are labelled in dark gray. Countries in light grey were not included in this analysis. The maps were created using mapchart.net and the basemap is available at: https://www.mapchart.net/world.html.

Twenty-eight countries (28/37, 75.7%) reported to be endemic for *B. abortus* in livestock (Fig 5), but research studies only confirmed the presence of this species in 4 countries (4/28, 14.3%) primarily in the Americas (2/4, 50%) followed by Africa (1/4, 25%) and Asia (1/4, 25%). Nineteen countries (19/37, 51.3%) reported to be endemic for *B. melitensis* in livestock, but research studies only confirmed the presence of this species in 2 countries (2/19; 10.5%) in Africa (1/2, 50%) and Asia (1/2, 50%). Interestingly, information was lacking regarding the reported presence of *B. melitensis* in Ecuador even though *B. melitensis* DNA was detected in goats [16]. Finally, 6 countries (6/37, 16.2%) reported to be endemic for *B. suis,* but research studies only identified this species in 1 country (1/6, 16.7%) located in the Americas. These results show that findings from research studies in accordance with WOAH guidelines mainly coincide with self-reported disease status at the country level.

## Discussion

Since the discovery of *Brucella* in 1887 by David Bruce, livestock brucellosis has been consistently classified among the most economically important zoonotic diseases under resource-limited settings [1,17]. For its effective control, adequate diagnosis is necessary. Diagnostic assays are pivotal during each step of disease control including the determination of disease prevalence, assessment of the efficacy of control and elimination practices (test and slaughter) as well as during surveillance to confirm eradication. Demand for diagnostic testing for livestock brucellosis continues to rise due to the high number of cases in resource limited regions as well as a poor understanding of the epidemiological status of the disease. Currently, a multitude of assays exist with variable complexity, cost, and performance [18]. This variability highlights the fact that brucellosis is a disease in which the interpretation of assays results and applicability can be influenced, not only by technical aspects and assay limitations, but by the equally important epidemiological context. Therefore, the understanding of assay advantages and limitations is extremely important. Currently, the diagnosis of livestock brucellosis is based on indirect or direct methods. Indirect methods focus on the detection of the humoral or cell-mediated immune response while direct approaches include bacteriological culture and direct detection of the organism’s genetic material via PCR. Even though PCR is described as a mean of detection and identification for *Brucella*, a standardized protocol is not yet described by the WOAH. In fact, the extensive number of protocols utilized in studies renders harmonization and standardization difficult in addition to the lack of proper validation of selected protocols [18]. Current methods aiming at determining immune responses against the pathogen are mainly based on the detection of IgM, IgG, and IgA antibodies against LPS, mostly the O-polysaccharide [19] in serum and milk. Alternatively, cell-mediated response has been utilized via the brucellin skin test which is based on a delayed-type hypersensitivity reaction induced at the site of inoculation following local injection of an LPS-free antigen observed in animals that have already been exposed to *Brucella*.

The extensive variety of available assays has led to numerous research studies with significant diversity in the diagnostic tests used and results obtained, generating the need for the publication of reviews and meta-analyses to compare and summarize research findings. Work has been done to provide an overview of the epidemiology of brucellosis in livestock in various regions of the world, mainly focusing on seroprevalence levels and molecular analysis of circulating *Brucella* species [20–22]. Here, we provide an overview of diagnostic tests used worldwide and compare methods used in each study against WOAH guidelines to assess the confidence level of research findings. Subsequently, data from studies performed in accordance with recommendations were compared to disease status reported by countries to determine the correlation between research findings and public information on brucellosis epidemiology worldwide.

In the present study, a total of 349 studies that reported data on brucellosis prevalence were retrieved and analyzed. Most of the studies were published between 2009 and 2023 (Fig 2). This is in accordance with the recent reemergence of the interest by many countries with the inclusion of brucellosis as a priority disease to implement improved control measures by local authorities [23–25]. This period also coincides with the apparition of predatory journals which drove the demand for research studies, independently of the quality of the study, higher [26]. However, only 2 (2/349, 0.6%) research studies included in this review were found to have been published in known predatory journals. Thus, the drastic increase in publication numbers was not due to this type of publication during this period. As expected, cattle were the most sampled of the livestock species, as they are the most common livestock species with over 1.3 billion animals globally and the main livestock production system worldwide (668 billion US dollars in 2022) [27,28]. Studies focusing on small ruminants known to carry *B. melitensis* were less represented with half the number of studies when compared to cattle. Indeed, small ruminant production (sheep and goats combined) is far less important than cattle, representing 110 billion US dollars in 2022 [28]. Finally, swine were significantly underrepresented with only 18 studies. This is striking as swine represent a significant agricultural asset for meat production in some of the European countries that are endemic for *B. suis* (such as Italy and Germany) as well as China, representing a total of 352 billion US dollars worldwide in 2022, demonstrating a significant gap in our current knowledge of the disease status in this species [28,29]. Lack of studies can be partially attributed to the challenging nature of diagnosis in pigs because of poor performance of immunological tests due to high levels of cross reactivity with other Gram-negative bacteria [30]. However, due to the emergence of the disease in feral swine populations and the threat this poses to swine farms, further investigation on this animal species should be encouraged [31,32]. Buffaloes are mainly kept for their adaptation for tropical climates, meat, milk, and draught power, especially in flooded fields and although not traditionally thought of as a main livestock species, were also included in the analysis since this animal species is known to carry *B. abortus* and represents a significant livestock production system (88 billion US dollars in 2022) specifically in Asia and the Americas with approximately 200 million animals [27,28]. Strikingly, more studies have been conducted in this species (51 vs 18) than in swine despite the fact that the swine population worldwide is significantly larger than buffaloes (916 million and 183 million, respectively) [27].

Research studies included in this work were globally distributed. Specifically, most of the studies were performed in Africa, followed by Asia, the Americas, and Europe. Interestingly, this distribution correlates with newly published data showing that Asia accounts for most of the human brucellosis cases while Africa has the most risk (cases per millions per year) [8]. As expected, no studies from Oceania were found due to the eradication of the disease in Australia and New-Zealand [33].

The socioeconomic status of each country was taken into consideration in this analysis to assess the impact of resources on the conduction of research studies. We found that almost half of the studies were performed in lower-middle income and low-income countries (171/349 and 83/349, respectively). Further analysis showed that studies focusing on resources limited countries were not funded by countries with higher resources but by countries of similar socioeconomic status (S4 Table). Furthermore, studies conducted by higher resource countries did not correlate with studies done in accordance with guidelines (S5 Table). This suggests that lack of resources does not correlates with a lower number of research studies being conducted or with less accordance with WOAH guidelines. When the precise location of studies was extracted, we observed that research was mainly carried out in rural areas. This was expected as livestock is usually the main activity and livelihood in rural areas demonstrating that research studies are conducted in high-risk areas.

One of our objectives was to provide an overview of diagnostic tests used for diagnosis of brucellosis in livestock worldwide. Strikingly, we observed 100 different combinations of indirect and direct diagnostic methods utilized in research studies clearly demonstrating a lack of standardization of diagnostic practices. Immunological approaches were employed in the majority of studies likely due to the ease of access of the required samples (serum and/or milk) and the less demanding experimental protocols when compared to bacteriological culture or molecular based techniques. Overall, RBT was found to be the most used assay (223/349), and this could be explained by its low cost and ease of use. Another advantage of agglutination tests such as RBT is the fact that they do not require a strict validation using local samples from target populations, making them easier to perform in the field [9]. In fact, the most used assays following RBT were iELISA (128/349), CFT (70/349), and bacteriological culture (68/349) which all require dedicated equipment and/or facilities, advanced training, and have a higher cost which would hinder their use in resources limited regions. However, these assays were all used in Africa and Asia where most low and lower-middle countries are located indicating that test availability is not the reason for the low level of adherence to guidelines. The majority of immunological assays are based on the use of lipopolysaccharide (LPS) from the outer membrane of *Brucella* as the antigen which can lead to cross reactions with other Gram-negative bacteria. For this reason, WOAH recommends that positive reactions by LPS-based assays be confirmed using complementary strategies. Following this logic, a number of studies used a combination of immunological assays. The most used combination was RBT followed by CFT.

To assess the level of confidence of epidemiological results reported worldwide, we analyzed experimental protocols followed in each study and compared them against guidelines published by the WOAH. While RBT was performed according to guidelines in the majority of studies that provided sufficient details on the experimental protocol followed, significant flaws were identified in the use of other tests. Specifically, regarding indirect and competitive ELISAs, validation and determination of the cut-off values using local samples from target populations was not performed (114/128, 89.1%). This is critical as immunological background of animals in different geographic regions can differ, possibly leading to biased prevalence results [34]. Therefore, guidelines recommend the revalidation of assays when deployed in different geographic regions [35]. Among other flaws identified in the use of immunological assays, we note the use of SAT on sheep and goat serum samples despite the mention in WOAH guidelines that SAT should be used in cattle only. Additionally, the use of 2-ME and Rivanol test as confirmatory was reported in 16 studies. Both assays are based on the addition of an agent that leads to the breakage of disulfide bonds present in antibodies thus inactivating the agglutinating activity of immunoglobulins. This is assumed to improve performance of the tests and limit false positive reactions, especially in dog and swine samples. However, these assays have shown to have a lower sensitivity and do not solve cross-reactivity issues [36,37]. 2-ME is used for the diagnosis of *B. canis* infection in dog samples but was used as a confirmatory test in cattle, buffaloes, sheep, and goats in 13 studies. While the use of Rivanol test is not mentioned in official guidelines, it has been used in swine samples to limit false positive reactions [19]. In this study, the Rivanol test was not considered to be in accordance with guidelines, but was used to confirm infection in cattle, buffaloes, sheep, and goats in 3 studies which is also contrary to recommendations found in the literature. When conducted appropriately, bacteriological culture is considered the gold standard for brucellosis diagnosis as it provides a definite proof of the presence of live organisms in samples. This is especially valuable during the acute phase of infection when animals show signs such as abortion when tissues with high bacterial load are made available. However, when focusing on herds or individual animals devoid of clinical signs, sensitivity of bacteriological culture depends on the method used such as the choice and number of samples taken, along with culture conditions and media selection [38]. Several studies used culture media and sample preparation practices not recommended for the isolation of *Brucella*. To ensure reliable results, samples used for bacteriological culture need to be selected and processed to maximize chances to encounter the pathogen which involves the homogenization of tissues using a stomacher or a similar device as the bacteria will be found inside cells. Several studies simply rubbed cuts of tissues onto media, minimizing the potential for isolation. Due to the slow growth of *Brucella* compared to bacteria commonly found in animal samples, the use of a selective media is essential and was overlooked in 5 studies. Selective media used in the isolation of *Brucella* from tissues samples contain various antibiotics and antifungal agents which limit the growth of contaminants from samples. One of the most commonly used is Farrell’s media which is recommended by the WOAH as it has been shown to be highly useful for the isolation of *Brucella* however, in recent years, it was shown that 2 antibiotics used in this medium (nalidixic acid and bacitracin) can limit the growth of *B. abortus*, *B. melitensis* and *B. ovis* to some extent [39]. Therefore, the WOAH recommends a complementary medium named CITA (composed of different antibiotic and antifungal agents) to be used alongside Farrell’s in order to increase diagnostic sensitivity [9,40]. Instead of using polymyxin B sulfate, bacitracin, natamycin, nalidixic acid and nystatin the CITA medium contains colistin methanesulfonate, nitrofurantoin, nystatin, and amphotericin B. During our analysis, no distinction was made between these two media, and both were considered as in accordance with guidelines. Although bacteriological culture is recognized as the gold standard for brucellosis diagnosis, its success is not guaranteed and depends heavily on the use of the appropriate media and conditions outlined in these guidelines as well as the stage of the infection and proper sample selection. Detection of *Brucella* can also be done using PCR based methods. Although mentioned in the WOAH guidelines, it is important to note that there are no standardized PCR methods for the diagnosis of brucellosis from tissues and further research efforts are required to develop a thoroughly validated PCR assay. The lack of standard methods to accurately detect *Brucella* in tissues samples is well described in the literature [41]. However, it is agreed that tissues most susceptible to high concentrations of the bacteria (such as lymph nodes) should be targeted to increase chances of DNA detection. Unfortunately, such tissues samples are usually only collected from slaughtered or deceased animals, making systematic collection challenging. In an effort to use PCR on more easily accessible samples, studies have attempted to develop protocols using blood and serum samples. However, research has shown that blood and serum contain components that inhibit PCR reaction which makes them improper samples to use, even though they are easily available [42,43]. Additionally, since *Brucella* is an intracellular pathogen, it would not be present in the serum which is devoid of cells, and animals are not always bacteremic, making the chances of detecting the pathogen in the blood even smaller [44]. Despite these limitations, PCR based methods are invaluable for the identification of *Brucella* species. They represent a significant step up from biochemical techniques as they are faster and less cumbersome even though biotyping is still recommend within the guidelines. However, the current and most commonly used approaches which include the Bruce Ladder and the AMOS PCR have shown some limitations in recent years to differentiate between *Brucella* species. For example, two newly identified *Brucella* species now known as *B. nosferapti* and *B. amazoniensis*, were initially classified as *B. suis* using the Bruce Ladder. However, further analysis refuted these results which lead to the discovery of 2 new *Brucella* species [4,45]. Regarding *B. nosferapti* its classification was resolved using MLVA-16 which showed that the isolate did not cluster with any other *Brucella* species [45]. Using whole genome sequencing (WGS), it was discovered that its closest phylogenetic relative was *B. amazoniensis*, a new species discovered earlier in French Guiana [4]. In this case, B. *amazoniensis* presented a pattern on the Bruce Ladder consistent with *B. suis*, *B. microti* and *B. neotomae*. Using WGS, it was observed that this isolate did not cluster with any other existing *Brucella* species and was identified as a new species. These examples show that, although rare, misclassification can occur. They also demonstrate the need to be familiar with the limitations of commonly used techniques and consider the use of new generation diagnostic techniques as a complement (if available) when such situations occur.

Besides the choice of a proper diagnostic test and the use of a recommended experimental protocol, careful interpretation of results is required to obtain accurate data. When carrying out multiple immunological assays, results should not be interpreted independently as they can widely vary depending on the sensitivity and specificity of each test. Unfortunately, this approach was used in 145 studies. When performing multiple immunological assays on a target population, guidelines suggest that results should be considered in relation to each other rather than independently. Series testing is based on the realization and interpretation of multiple tests in a specific order. During this process, a first assay is applied to all samples. Then, samples that produced a positive signal are further analyzed using a second test. Samples that produced a positive result using both assays are classified as positive. This approach leads to a higher specificity and an increased confidence in the results. This approach was used in 151 of the studies. Another possible approach is the simultaneous use of 2 immunological assays on all samples or “parallel testing”. With this methodology, samples are regarded as positive if they produced a positive result to 1 of the 2 immunological tests, favoring sensitivity and increasing chances of correctly identifying infected animals. This approach was used in 14 studies. These results highlight that on top of flaws in the experimental protocols used, interpretation of results was also a key parameter overlooked by research studies possibly leading to inaccurate data being published. Another critical issue regarding the interpretation of tests results was the conclusion of the presence of a specific *Brucella* species in studies where only immunological approaches were performed. Indeed, use of LPS as the antigen makes distinction between smooth strains (*B. abortus*, *B. melitensis*, and *B. suis*) impossible. In our study, we found 7 (7/349, 2%) studies that made such an unsustained claim. These conclusions might arise from the belief that *Brucella* species cannot infect other animal species other than their preferred host (i.e., cattle for *B. abortus*, small ruminants for *B. melitensis*, and swine for *B. suis*). Following this logic, several studies claimed that because the infected animal was a cow it must be infected with *B. abortus*. However, such claims cannot be made without further characterization of the species by molecular approaches as *B. melitensis* can spillover and infect cattle, and *B. abortus* can be found in small ruminants [46–48]. Therefore, direct methods are of critical importance for the identification of *Brucella* species.

Following the analysis of individual parameters including selection and preparation of samples, choice of diagnostics tests, experimental protocols used, validation of the assay when appropriate, and interpretation, overall accordance to guidelines was determined. Shockingly, we found that in most studies (73.6%), 1 or several of the above-mentioned parameters were not conducted according to recommendations. Accordance with guidelines was not impacted by socioeconomic status of countries, making this a global issue. These results indicate that the majority of data available on the diagnosis of brucellosis in livestock originate from studies with significant flaws, questioning the veracity of reported findings, and leading to a false sense of understanding of the global brucellosis situation in livestock.

The WOAH identifies brucellosis as a reportable disease, and member countries are expected to report the presence or absence of *B. melitensis*, *abortus,* and *suis* in livestock every year. This information is then posted on the WAHIS interface which is accessible to the public [25]. The major issue with country level reports is that they do not include details on the diagnostic methods used. This makes comparison with available guidelines impossible. We have demonstrated that the majority of studies possess significant flaws that could lead to the diffusion of inaccurate prevalence data. We can hypothesize that such data could be used by countries as a basis for annual reports, possibly leading to a false picture of brucellosis epidemiology in livestock. Furthermore, these possibly incorrect and publicly available reports can be used by governments and policy makers to target efforts and resources based on a false picture of brucellosis epidemiology in livestock. To assess the quality of reported data, we focused on research studies conducted in agreement with guidelines and compared their findings with self-reported disease status of countries. By doing so, we found a very small number of research studies (from a small handful of countries) in agreement with guidelines corroborating the presence of *B*. *abortus*, *B. melitensis*, and *B. suis,* highlighting the lack of research data available to support the self-reported disease status to the WOAH. Furthermore, most studies done in accordance with WOAH were only based on immunological data which does not allow for discrimination between the different species. However, as countries only report the presence or absence of a given species, we can hypothesize that without isolation and identification of the circulating species, countries may report the presence of a certain species based on seroprevalence data only.

Unfortunately, we were not able to retrieve all identified articles and our review was also limited to those published in French and English. Numerous studies were published in Portuguese, mainly focusing on Brazil, and several studies published in Chinese were also not included due to the language barrier. Another limitation was the lack of methodology details in a large portion of studies which prevented us from assessing if guidelines were correctly followed. Consequently, these studies were classified as having insufficient information and could not be used to draw any conclusions. Finally, due to a numerous lack of countries with studies conducted according to WOAH guidelines, generalizable conclusions per regions were difficult to ascertain.

## Conclusions

In conclusion, our analysis revealed that only a small fraction of published scientific studies focusing on the diagnosis of brucellosis in livestock are in accordance with the guidelines given by the WOAH. Even if challenged, guidelines found in the terrestrial manual are not strict rules but provide a framework to ensure proper diagnosis and reliable data [38]. The main issue contributing to the low number of accurate studies, leading to significant knowledge gaps concerning the presence and prevalence of brucellosis in livestock, does not seem to necessarily be the availability of diagnostics tests or the ability of researchers to properly conduct them, but rather the unawareness or misinterpretation of these publicly available guidelines. Initiatives should be pursued to enhance capacity building and education towards the use of diagnostic assays and their accurate interpretation using the WOAH guidelines as a reference. Further efforts should be made to ensure the proper design of scientific studies to produce reliable data and minimize further loss of limited resources including time, effort, and money. Complicated tests or extensive equipment are not required to accurately diagnose brucellosis in livestock. One simple and accessible assay such as the Rose Bengal test offers confidence in results when used correctly in a well-designed diagnostic strategy allowing for screening of high numbers of animals. However, it is an immunological assay, and the main drawback remains its inability to identify the *Brucella* species infecting animals. As a zoonotic disease, the accurate diagnosis of brucellosis in livestock following WOAH guidelines is essential to ensure timely control of the disease, help prevent human infections and reduce economic losses due to unnecessary culling of healthy animals. This work reveals a lack of research studies done in accordance with these guidelines which limits a proper understanding of the epidemiological situation of brucellosis worldwide. Moreover, the lack of research findings limits our ability to corroborate brucellosis disease status reported by individual countries to the WOAH. These findings challenge our current understanding of brucellosis worldwide possibly leading to erroneous estimates of brucellosis cases in livestock.

## Materials and methods

This study was performed according to the Preferred Reporting Items in Systematic Reviews and Meta-Analyses (PRISMA) guidelines (S1 and S2 Checklists) [49].

### Search strategy

On August 28, 2023, a search was conducted utilizing the following databases: PubMed, Embase, and Web of Science. Keywords were investigated within title and abstract fields without restriction of time or country. The complete list of search terms and search strategy are provided in the additional material (S1 Fig). For this study, PICO (population, intervention, comparison, and outcome) included 1) population: cattle, buffaloes, sheep, goats, and pigs, 2) intervention: diagnostic test(s), 3) comparison: none, and 4) outcome: seroprevalence or isolation and identification of the pathogen.

### Selection of the studies

Study selection and eligibility assessment were completed using Covidence. All retrieved articles were imported into Covidence, and duplicates were either automatically or manually removed. Titles and abstracts of uploaded articles were then screened by two independent reviewers (SV and CGL) for their relevance to the study objectives and inclusion/exclusion criteria. Subsequently, conflicts were resolved by consensus. Finally, the relevant articles were subjected to independent full text review (SV and CGL) before their inclusion in the study.

### Eligibility criteria

Studies with the following characteristics were included within this review: cross-sectional seroprevalence or molecular typing and identification that incorporated cattle, buffaloes, small ruminants, and/or swine, and communicated *Brucella* diagnostics, written in English or French. French was also used in the search since it is the official language of several Central and West African countries in which brucellosis is believed to be endemic. Studies excluded from the review were those focusing on 1) *Brucella* species other than *Brucella melitensis*, *abortus,* or *suis*, 2) wildlife or animal species that differ from the ones mentioned above, along with 3) those focusing on the development or validation of diagnostic tests, and 4) those focusing only on human brucellosis. Original research articles describing cross-sectional studies were included whereas reviews, case reports, and case-control studies were excluded. The complete list of exclusion and inclusion criteria are provided in the additional material (S2 Fig).

### Data extraction and quality assessment

A data spreadsheet was designed that included: year, geographic location, national economic status, study population, sample size, sample type, diagnostic test performed, diagnostic methodology, result interpretation, identified *Brucella* species (if identification was performed), adherence to publicly available WOAH guidelines, and results. Articles were divided between the reviewers for data extraction. Data from all studies were checked for accuracy by both reviewers (SV, CGL). When necessary, discrepancies among the reviewers were resolved by consensus. All studies were included in analysis. Data spreadsheet is provided in the additional material (S1 Appendix).

Assessment of agreement with WOAH guidelines for each study was performed by comparing sample type, sample handling and/or collection, experimental protocol of diagnostic assay (specifically, the experimental protocol and conditions for immunological assays, kits and antigen used for indirect and competitive ELISAs, primers and amplification conditions for PCR-based methods, culture media and sample preparation for bacteriological culture), validation using local samples (specifically for indirect and competitive ELISAs), and result interpretation against WOAH guidelines found in the WOAH Manual of Diagnostic Test and Vaccines for Terrestrial Animals [9].

### Socioeconomic status

Gross National Income (GNI) per capita was extracted from the World Bank databank [10]. Countries were grouped by lending group according to the World Bank Atlas method [11]. Specifically, low-income economies are countries with a GNI per capita of $1,135 or less in 2022; lower middle-income economies are those with a GNI per capita between $1,136 and $4,465; upper middle-income economies are those with a GNI per capita between $4,466 and $13,845; high-income economies are those with a GNI per capita of $13,846 or more [50].

## Supporting information

S1 Fig Overview of search terms and strategy by database. (DOCX)

S2 Fig Inclusion and exclusion criteria. (DOCX)

S3 Fig Schematic representation of the treatment of data extracted from the included studies. (A) Individual countries from each study were assigned into a specific socioeconomic group based on the World Bank dataset. Country was also used to classify studies by continent. (B) Based on the precise location of the study, the geographic area (urban, rural, or both) was determined. If the specific country or the subregion was not disclosed, the status was determined as undefined. (C) From each study, the diagnostic test used was extracted and the protocol analyzed. If the protocol was not performed according to the WOAH guidelines, if the samples were not appropriate, or if the testing strategy not in accordance with guidelines, the study was classified as not in accordance. If not enough information was provided, the study was classified as insufficient information. If the diagnostic assay, selected samples, and testing strategy followed the WOAH guidelines, the study was classified as in accordance.
(DOCX)

S1 Table Geographical distribution of studies by animal species. (DOCX)

S2 Table Classification of PCR protocols utilized in research studies to determine *Brucella* species with respect to agreement with recommendations made by the WOAH. (DOCX)

S3 Table Correlation between findings of research studies in accordance with WOAH guidelines published between 2014 and 2018 and disease status reported by countries to WOAH. (DOCX)

S4 Table Relationship between socioeconomic status of country of institution and of the country where the study was performed. (DOCX)

S5 Table Accordance to WOAH guidelines depending on socioeconomic status of leading institution of studies published between 2009 and 2023. (DOCX)

S1 Checklist PRISMA 2020 abstract checklist for systematic reviews. (DOCX)

S2 Checklist RISMA 2020 checklist for systematic reviews. (DOCX)

S1 Appendix Extracted data. (XLSX)
