# Supplementary material for: What do we really know about brucellosis diagnosis in livestock worldwide? A systematic review
Source: PLoS Negl Trop Dis. 2025 Jun 17;19(6):e0013185. doi: 10.1371/journal.pntd.0013185 (PMC12173231; doi:10.1371/journal.pntd.0013185)
Supplement: S1 Fig — (DOCX) [file pntd.0013185.s001.docx]

**S1 Fig. Overview of search terms and strategy by database.**

**Database (Platform): Date Searched**

PubMed (U.S. National Library of Medicine, National Institutes of Health): August 28, 2023

**Title and Abstract Only**

| **Search** | **Query** | **Number of Results** |
| --- | --- | --- |
| 1 | (Brucella OR Brucellosis OR Brucellose OR “B. melitensis” OR “Brucella melitensis” OR “B. abortus” OR “Brucella abortus” OR “B. suis” OR “Brucella suis” OR “B. canis” OR “Brucella canis” OR “Malta fever” OR “Fièvre de Malte” OR “undulant fever” OR “fièvre ondulante”) | 20,130 |
| 2 | (diagnostic OR diagnos* OR detection OR détection OR analysis OR analyse OR surveillance OR screening OR dépistage OR identification OR monitoring OR “parallel testing” OR “test en parallele” OR “series testing” OR “test en serie” OR incidence OR prevalence OR prévalence OR assay OR serological OR sérologique OR bacteriological OR bactériologique) | 10,805,458 |
| 3 | ((culture OR isolation OR isolement OR “bacteriological culture” OR “bacterial culture” OR “culture bactérienne” OR “culture bactériologique”) OR (PCR OR “polymerase chain reaction” OR IS711 OR qPCR OR “real-time PCR” OR “RT-PCR” OR “Bruce ladder” OR “Suis ladder” OR “réaction de polymérisation en chaîne” OR “PCR en temps reel” OR “PCR quantitative”) OR (“Rose Bengal test” OR “Rose Bengal” OR RBT OR “Rose Bengale”) OR (“Buffered Brucella antigen” OR BBAT) OR (“Fluorescence polarization assay” OR FPA OR “fluorescence polarization” OR “Fluorescence polarisation assay” OR “fluorescence polarization” OR “polarisation de fluorescence”) OR (“Complement fixation test” OR CFT OR “complement fixation” OR “test de fixation du complement”) OR (“Enzyme linked immunosorbent assay” OR ELISA OR i-ELISA OR “indirect ELISA” OR c-ELISA OR “competitive ELISA” OR “indirect Milk ELISA” OR “milk ELISA” OR “dosage immuno-enzymatique” OR “dosage enzymatique”) OR (“Brucellin skin” OR “brucellin skin test” OR BST) OR (“Serum agglutination test” OR SAT OR agglutination OR “test d’agglutination”) OR (“Rapid slide agglutination” OR RSAT OR 2ME-RSAT) OR (“Buffered Plate agglutination test” OR BPAT) OR (“Milk ring test” OR MRT OR “test de l’anneau de lait”) OR (“agar gel immunodiffusion test” OR AGID OR “agar gel immunodiffusion’ OR “agar immunodiffusion”) OR (“Lateral flow assay” OR “LFA” OR “Lateral Flow Immunochromatography Assay” OR “LFIA” OR “test antigénique” OR “test immuno-chromatographique” OR “test immunochromatographique”)) | 1,928,964 |
| 4 | #1 AND #2 AND #3 | **4,086** |
| 5 | #1 AND #2 | 8,958 |
| 6 | #1 AND #3 | 5,428 |

Used <https://sr-accelerator.com/#/polyglot> to translate PubMed search terms in other databases.

**Database (Platform): Date Searched**

Embase (OVID): August 28, 2023

**Title and abstract search.**

| **Search** | **Query** | **Number of Results** |
| --- | --- | --- |
| 1 | (Brucella OR Brucellosis OR Brucellose OR “B. melitensis” OR “Brucella melitensis” OR “B. abortus” OR “Brucella abortus” OR “B. suis” OR “Brucella suis” OR “B. canis” OR “Brucella canis” OR “Malta fever” OR “Fièvre de Malte” OR “undulant fever” OR “fièvre ondulante”) | 19286 |
| 2 | (diagnostic or diagnos* or detection or analysis or analyse or surveillance or screening or depistage or identification or monitoring or "parallel testing" or "test en parallele" or "series testing" or "test en serie" or incidence or prevalence or assay or serological or serologique or bacteriological or bacteriologique) | 17979887 |
| 3 | ((culture OR isolation OR isolement OR "bacteriological culture" OR "bacterial culture" OR "culture bactérienne" OR "culture bactériologique") OR (PCR OR "polymerase chain reaction" OR IS711 OR qPCR OR "real-time PCR" OR RT-PCR OR "Bruce ladder" OR "Suis ladder" OR "réaction de polymérisation en chaîne" OR "PCR en temps reel" OR "PCR quantitative") OR ("Rose Bengal test" OR "Rose Bengal" OR RBT OR "Rose Bengale") OR ("Buffered Brucella antigen" OR BBAT) OR ("Fluorescence polarization assay" OR FPA OR "fluorescence polarization" OR "Fluorescence polarisation assay" OR "fluorescence polarisation" OR "polarisation de fluorescence") OR ("Complement fixation test" OR CFT OR "complement fixation" OR "test de fixation du complément") OR ("Enzyme linked immunosorbent assay" OR ELISA OR i-ELISA OR "indirect ELISA" OR c-ELISA OR "competitive ELISA" OR "Milk indirect ELISA" OR "milk ELISA" OR "dosage immuno-enzymatique" OR "dosage enzymatique") OR ("Brucellin skin" OR "brucellin skin test" OR BST) OR ("Serum agglutination test" OR SAT OR agglutination OR "test d’agglutination") OR ("Rapid slide agglutination" OR RSAT OR 2ME-RSAT) OR ("Buffered Plate agglutination test" OR BPAT) OR ("Milk ring test" OR MRT OR "test de l’anneau de lait") OR ("agar gel immunodiffusion test" OR AGID OR "agar gel immunodiffusion" OR "agar immunodiffusion") OR ("Lateral flow assay" OR LFA OR "Lateral Flow Immunochromatography Assay" OR LFIA OR "test antigénique" OR "test immuno-chromatographique" OR "test immunochromatographique")) | 4006050 |
| 4 | 1 AND 2 AND 3 | **7517** |
| 5 | #1 AND #2 | 12973 |
| 6 | #1 AND #3 | 9042 |

**Database (Platform): Date Searched**

Web Of Science: August 28, 2023

**Title and Abstract Search**

| **Search** | **Query** | **Number of Results** |
| --- | --- | --- |
| 1 | (Brucella OR Brucellosis OR Brucellose OR "B. melitensis" OR "Brucella melitensis" OR "B. abortus" OR "Brucella abortus" OR "B. suis" OR "Brucella suis" OR "B. canis" OR "Brucella canis" OR "Malta fever" OR "Fièvre de Malte" OR "undulant fever" OR "fièvre ondulante") | 18,410 |
| 2 | (diagnostic OR diagnos* OR detection OR analysis OR analyse OR surveillance OR screening OR depistage OR identification OR monitoring OR "parallel testing" OR "test en parallele" OR "series testing" OR "test en serie" OR incidence OR prevalence OR assay OR serological OR serologique OR bacteriological OR bacteriologique) | 22,509,019 |
| 3 | ((culture OR isolation OR isolement OR "bacteriological culture" OR "bacterial culture" OR "culture bactérienne" OR "culture bactériologique") OR (PCR OR "polymerase chain reaction" OR IS711 OR qPCR OR "real-time PCR" OR RT-PCR OR "Bruce ladder" OR "Suis ladder" OR "réaction de polymérisation en chaîne" OR "PCR en temps reel" OR "PCR quantitative") OR ("Rose Bengal test" OR "Rose Bengal" OR RBT OR "Rose Bengale") OR ("Buffered Brucella antigen" OR BBAT) OR ("Fluorescence polarization assay" OR FPA OR "fluorescence polarization" OR "Fluorescence polarisation assay" OR "fluorescence polarisation" OR "polarisation de fluorescence") OR ("Complement fixation test" OR CFT OR "complement fixation" OR "test de fixation du complément") OR ("Enzyme linked immunosorbent assay" OR ELISA OR i-ELISA OR "indirect ELISA" OR c-ELISA OR "competitive ELISA" OR "Milk indirect ELISA" OR "milk ELISA" OR "dosage immuno-enzymatique" OR "dosage enzymatique") OR ("Brucellin skin" OR "brucellin skin test" OR BST) OR ("Serum agglutination test" OR SAT OR agglutination OR "test d’agglutination") OR ("Rapid slide agglutination" OR RSAT OR 2ME-RSAT) OR ("Buffered Plate agglutination test" OR BPAT) OR ("Milk ring test" OR MRT OR "test de l’anneau de lait") OR ("agar gel immunodiffusion test" OR AGID OR "agar gel immunodiffusion" OR "agar immunodiffusion") OR ("Lateral flow assay" OR LFA OR "Lateral Flow Immunochromatography Assay" OR LFIA OR "test antigénique" OR "test immuno-chromatographique" OR "test immunochromatographique")) | 3,106,692 |
| 4 | #1 AND #2 AND #3 | **4,530** |
| 5 | 1 AND 2 | 9,210 |
| 6 | 1 AND 3 | 5,631 |
