# Supplementary material for: What do we really know about brucellosis diagnosis in livestock worldwide? A systematic review
Source: PLoS Negl Trop Dis. 2025 Jun 17;19(6):e0013185. doi: 10.1371/journal.pntd.0013185 (PMC12173231; doi:10.1371/journal.pntd.0013185)
Supplement: S2 Fig — (DOCX) [file pntd.0013185.s002.docx]

**S2 Fig. Inclusion and exclusion criteria.**

Inclusion criteria:

- All countries
- All years
- *Brucella melitensis*, *Brucella abortus* or *Brucella suis*.
- Seroprevalence or isolation and identification of *Brucella*
- Livestock species: cattle, buffalo, sheep, goats, or swine
- Studies in English and French

Exclusion criteria :

- Development or validation of diagnostic tests
- Determination of analytical sensitivity and specificity of diagnostic tests
- Wildlife or other animal species that are not described in inclusion criteria
- Vaccination
- Cheese and milk products only
- Other *Brucella* species than the ones listed in inclusion criteria
- Reviews, case reports and case-control studies
- Full text not available
