# Supplementary material for: What do we really know about brucellosis diagnosis in livestock worldwide? A systematic review
Source: PLoS Negl Trop Dis. 2025 Jun 17;19(6):e0013185. doi: 10.1371/journal.pntd.0013185 (PMC12173231; doi:10.1371/journal.pntd.0013185)
Supplement: S3 Fig — (A) Individual countries from each study were assigned into a specific socioeconomic group based on the World Bank dataset. Country was also used to classify studies by continent. (B) Based on the precise location of the study, the geographic area (urban, rural, or both) was determined. If the specific country or the subregion was not disclosed, the status was determined as undefined. (C) From each study, the diagnostic test used was extracted and the protocol analyzed. If the protocol was not performed according to the WOAH guidelines, if the samples were not appropriate, or if the testing strategy not in accordance with guidelines, the study was classified as not in accordance. If not enough information was provided, the study was classified as insufficient information. If the diagnostic assay, selected samples, and testing strategy followed the WOAH guidelines, the study was classified as in accordance. (DOCX) [file pntd.0013185.s003.docx]

**S3 Fig. Schematic representation of the treatment of data extracted from the included studies.** (A) Individual countries from each study were assigned into a specific socioeconomic group based on the World Bank dataset. Country was also used to classify studies by continent. (B) Based on the precise location of the study the geographic area (urban, rural or both) was determined. If the specific country or the subregion was not disclosed, the status was determined as undefined. (C) From each study the diagnostic test used was extracted and the protocol analyzed. If the protocol was not performed according to the WOAH guidelines, the samples were not appropriate, or the testing strategy not in accordance with guidelines, the study was classified as not in accordance. If not enough information was provided the study was classified as insufficient information. If the diagnostic test, selected samples, and testing strategy followed the WOAH guidelines the study was classified as in accordance.
