# Supplementary material for: What do we really know about brucellosis diagnosis in livestock worldwide? A systematic review
Source: PLoS Negl Trop Dis. 2025 Jun 17;19(6):e0013185. doi: 10.1371/journal.pntd.0013185 (PMC12173231; doi:10.1371/journal.pntd.0013185)
Supplement: S1 Table — (DOCX) [file pntd.0013185.s004.docx]

|  | **Cattle** | **Buffalo** | **Sheep** | **Goat** | **Pig** |
| --- | --- | --- | --- | --- | --- |
| **Africa** | 131 | 2 | 52 | 59 | 5 |
| **Americas** | 15 | 8 | 4 | 10 | 3 |
| **Asia** | 82 | 40 | 50 | 46 | 5 |
| **Europe** | 4 | 1 | 4 | 4 | 5 |
| **Global** | 232 | 51 | 110 | 119 | 18 |

**S1 Table. Geographical distribution of studies by animal species.**
