# Supplementary material for: What do we really know about brucellosis diagnosis in livestock worldwide? A systematic review
Source: PLoS Negl Trop Dis. 2025 Jun 17;19(6):e0013185. doi: 10.1371/journal.pntd.0013185 (PMC12173231; doi:10.1371/journal.pntd.0013185)
Supplement: S2 Table — (DOCX) [file pntd.0013185.s005.docx]

**S2 Table. Classification of PCR protocols utilized in research studies to determine *Brucella* species with respect to agreement with recommendations made by the WOAH.**

| **Type of PCR and primers used** | **Performed according to WOAH recommendations** | **Not performed according to WOAH recommendations** | **Insufficient information** | **Total number of publications** |
| --- | --- | --- | --- | --- |
| Conventional AMOS PCR | 15 | 1 | 0 | 16 |
| Isolates | 14 | 0 | 0 | 14 |
| Serum | 0 | 1 | 0 | 1 |
| Tissues | 1 | 0 | 0 | 1 |
| Conventional Bruce Ladder | 7 | 0 | 0 | 7 |
| Isolates | 7 | 0 | 0 | 7 |
| Other samples | 0 | 0 | 0 | 0 |
| Both conventional AMOS PCR and Bruce Ladder | 3 | 0 | 0 | 3 |
| Isolates | 3 | 0 | 0 | 3 |
| Other samples | 0 | 0 | 0 | 0 |
