# Supplementary material for: What do we really know about brucellosis diagnosis in livestock worldwide? A systematic review
Source: PLoS Negl Trop Dis. 2025 Jun 17;19(6):e0013185. doi: 10.1371/journal.pntd.0013185 (PMC12173231; doi:10.1371/journal.pntd.0013185)
Supplement: S3 Table — (DOCX) [file pntd.0013185.s006.docx]

**S3 Table. Correlation between findings of research studies in accordance with WOAH guidelines published between 2014 and 2018 and disease status reported by countries to WOAH.**

|  | | | ***Brucella* species identified in research studies following WOAH guidelines** | | | **Disease status report to the WOAH** | | |
| --- | --- | --- | --- | --- | --- | --- | --- | --- |
|  | **Number of research studies published between 2014 and 2018** | **Number of research studies in accordance with WOAH guidelines** | *B. abortus* | *B. melitensis* | *B. suis* | *B. abortus* | *B. melitensis* | *B. suis* |
| **Africa** | **47** | **7** | **1** | **2** | **0** |  |  |  |
| Algeria | 2 | 2 | 1 | 2 | 0 | Endemic | Endemic | Insufficient Information |
| Cameroon | 2 | 0 | 0 | 0 | 0 | Disease Free | Insufficient Information | Insufficient Information |
| Côte d’Ivoire | 1 | 0 | 0 | 0 | 0 | Insufficient Information | Insufficient Information | Insufficient Information |
| Egypt | 3 | 0 | 0 | 0 | 0 | Disease Free | Endemic | Disease Free |
| Ethiopia | 7 | 3 | 0 | 0 | 0 | Endemic | Disease Free | Disease Free |
| Gambia | 1 | 0 | 0 | 0 | 0 | Insufficient Information | Insufficient Information | Insufficient Information |
| Guinea | 1 | 1 | 0 | 0 | 0 | Insufficient Information | Insufficient Information | Insufficient Information |
| Libya | 1 | 0 | 0 | 0 | 0 | Disease Free | Endemic | Disease Free |
| Namibia | 1 | 1 | 0 | 0 | 0 | Endemic | Disease Free | Disease Free |
| Nigeria | 14 | 0 | 0 | 0 | 0 | Endemic | Disease Free | Insufficient Information |
| Rwanda | 1 | 0 | 0 | 0 | 0 | Endemic | Endemic | Disease Free |
| South Africa | 1 | 0 | 0 | 0 | 0 | Endemic | Endemic | Disease Free |
| South Sudan | 1 | 0 | 0 | 0 | 0 | Disease Free | Insufficient Information | Insufficient Information |
| Sudan | 2 | 0 | 0 | 0 | 0 | Endemic | Disease Free | Disease Free |
| Tanzania | 3 | 0 | 0 | 0 | 0 | Endemic | Insufficient Information | Insufficient Information |
| Tunisia | 1 | 0 | 0 | 0 | 0 | Endemic | Endemic | Disease Free |
| Uganda | 5 | 0 | 0 | 0 | 0 | Endemic | Endemic | Disease Free |
| **Asia** | **35** | **2** | **1** | **2** | **0** |  |  |  |
| China | 3 | 0 | 0 | 0 | 0 | Endemic | Endemic | Endemic |
| India | 9 | 0 | 0 | 0 | 0 | Endemic | Insufficient Information | Insufficient Information |
| Indonesia | 1 | 0 | 0 | 0 | 0 | Endemic | Insufficient Information | Insufficient Information |
| Iran | 5 | 2 | 1 | 2 | 0 | Endemic | Endemic | Disease Free |
| Iraq | 1 | 0 | 0 | 0 | 0 | Endemic | Endemic | Disease Free |
| Lao PDR | 1 | 0 | 0 | 0 | 0 | Insufficient Information | Insufficient Information | Insufficient Information |
| Pakistan | 7 | 0 | 0 | 0 | 0 | \| Endemic \| \| --- \| | Endemic | Insufficient Information |
| Sri Lanka | 1 | 0 | 0 | 0 | 0 | Endemic | Disease Free | Disease Free |
| Tajikistan | 3 | 0 | 0 | 0 | 0 | Endemic | Endemic | Disease Free |
| Thailand | 1 | 0 | 0 | 0 | 0 | Endemic | Endemic | Disease Free |
| Turkey | 3 | 0 | 0 | 0 | 0 | Endemic | Endemic | Insufficient Information |
| **The Americas** | **17** | **0** | **2** | **1** | **1** |  |  |  |
| Argentina | 1 | 1 | 1 | 0 | 0 | Endemic | Endemic | Endemic |
| Brazil | 9 | 0 | 0 | 0 | 0 | Endemic | Disease Free | Disease Free |
| Colombia | 2 | 0 | 0 | 0 | 0 | Endemic | Disease Free | Disease Free |
| Ecuador | 3 | 1 | 1 | 1 | 1 | Endemic | Insufficient Information | Endemic |
| El Salvador | 1 | 0 | 0 | 0 | 0 | Endemic | Insufficient Information | Insufficient Information |
| Mexico | 1 | 0 | 0 | 0 | 0 | Endemic | Endemic | Disease Free |
| **Europe** | **4** | **0** | **0** | **0** | **0** |  |  |  |
| Croatia | 1 | 0 | 0 | 0 | 0 | Disease Free | Endemic | Endemic |
| Italy | 2 | 0 | 0 | 0 | 0 | Endemic | Endemic | Endemic |
| Spain | 1 | 0 | 0 | 0 | 0 | Endemic | Endemic | Endemic |
