# Supplementary material for: What do we really know about brucellosis diagnosis in livestock worldwide? A systematic review
Source: PLoS Negl Trop Dis. 2025 Jun 17;19(6):e0013185. doi: 10.1371/journal.pntd.0013185 (PMC12173231; doi:10.1371/journal.pntd.0013185)
Supplement: S4 Table — (DOCX) [file pntd.0013185.s007.docx]

|  | **Socioeconomic status of studied country** | | | | | | |
| --- | --- | --- | --- | --- | --- | --- | --- |
| **Socioeconomic status of country of last author's institution** | High income | Low income | Lower middle income | Undefined | Upper middle income | Grand Total |  |
| High | 10 | 13 | 29 | 0 | 3 | 55 |  |
| High/Low | 0 | 6 | 0 | 0 | 0 | 6 |  |
| High/Lower middle | 2 | 0 | 16 | 0 | 0 | 18 |  |
| Low | 0 | 41 | 1 | 0 | 0 | 42 |  |
| Low/ High/ Upper middle | 0 | 2 | 0 | 0 | 0 | 2 |  |
| Low/Upper middle | 0 | 2 | 0 | 0 | 0 | 2 |  |
| Low/Upper middle/Lower middle | 0 | 0 | 1 | 0 | 0 | 1 |  |
| Lower middle | 0 | 3 | 99 | 1 | 0 | 103 |  |
| Undefined | 0 | 0 | 1 | 1 | 0 | 2 |  |
| Upper middle | 0 | 3 | 4 | 0 | 62 | 69 |  |
| Upper middle/ High | 0 | 0 | 1 | 0 | 3 | 4 |  |
| Upper middle/ High/ Lower middle | 0 | 0 | 2 | 0 | 0 | 2 |  |
| Upper middle/Lower middle | 0 | 0 | 2 | 0 | 0 | 2 |  |
| Grand Total | 12 | 71 | 157 | 2 | 68 | 308 |  |

**S4 Table. Relationship between socioeconomic status of country of institution and of country where the study was performed.**
