# Supplementary material for: What do we really know about brucellosis diagnosis in livestock worldwide? A systematic review
Source: PLoS Negl Trop Dis. 2025 Jun 17;19(6):e0013185. doi: 10.1371/journal.pntd.0013185 (PMC12173231; doi:10.1371/journal.pntd.0013185)
Supplement: S5 Table — (DOCX) [file pntd.0013185.s008.docx]

|  | **Accordance with WOAH guidelines** | | | |
| --- | --- | --- | --- | --- |
| **Socioeconomic status of country of last author's institution** | **Insufficient information** | **No** | **Yes** | **Total** |
| High | 5 | 44 | 6 | 55 |
| High/Low | 0 | 5 | 1 | 6 |
| High/Lower middle | 1 | 17 | 0 | 18 |
| Low | 6 | 18 | 18 | 42 |
| Low/ High/ Upper middle | 0 | 2 | 0 | 2 |
| Low/Upper middle | 0 | 1 | 1 | 2 |
| Low/Upper middle/Lower middle | 0 | 1 | 0 | 1 |
| Lower middle | 6 | 88 | 9 | 103 |
| Undefined | 0 | 2 | 0 | 2 |
| Upper middle | 10 | 44 | 15 | 69 |
| Upper middle/ High | 0 | 4 | 0 | 4 |
| Upper middle/ High/ Lower middle | 0 | 2 | 0 | 2 |
| Upper middle/Lower middle | 0 | 1 | 1 | 2 |
| Grand Total | 28 | 230 | 51 | 309 |

**S5 Table. Accordance to WOAH guidelines depending on socioeconomic status of leading institution of studies published between 2009 and 2023.**
